# Prevalence of dental caries and associated factors among 12 years old students in Eritrea

**DOI:** 10.1186/s12903-017-0465-3

**Published:** 2017-12-29

**Authors:** Amanuel Kidane Andegiorgish, Bizen Weldmicheal Weldemariam, Meron Mehari Kifle, Filmon Gebreysus Mebrahtu, Henos Keflom Zewde, Micheal Gebregziabhir Tewelde, Mohammed Anwar Hussen, Winta Kesete Tsegay

**Affiliations:** 1 School of Public Health, Asmara College of Health Sciences, Asmara, Eritrea; 2 Ministry of Health, Asmara, Eritrea

**Keywords:** Dental caries, Dental health for children, Dental health services, Asmara, Eritrea

## Abstract

**Background:**

Dental caries is one of the most prevalent diseases of childhood in developing countries. However, there is a paucity of epidemiological data on the prevalence and associated factors of dental caries in Eritrea. The objective of this study was to assess the prevalence and associated factors of dental caries among 12 years old school children in Eritrea.

**Methods:**

A school based cross sectional study was conducted among 225 twelve years old students in two selected schools. One school from randomly selected urban and rural subzones of the country were selected. WHO adopted questionnaire and a standard checklist were used to collect relevant data. To assess dental caries, two examiners were calibrated by a certified dentist and inter observer agreement was calculated using the Cohen’s Kappa statistic (0.82). All data analysis was done using SPSS version 20.

**Results:**

The prevalence of dental caries was 78%, without significant difference between males (78%) and females (79%).The mean DMFT value was 2.50 (±2.21). The decayed component contributed 98.3% of the score as it had 2.44 (±1.2) share to the mean DMFT value. The first molar was the most affected tooth with a DMFT value of 1.55 (±1.36). The mean significant caries index score (SiC) was 4.97 (±1.9) which is higher than the upper limit of SiC value of 3 set by the WHO as a global average. More than half of the respondents had never visited a dentist and out of the students who had utilized a dental health facility, 82% of visits were due to dental pain while visits for regular checkups were cited by only 6.6% of the respondents.

**Conclusion:**

Dental caries was found to be a common public health problem among 12 years old Eritrean students. The prevalence of dental caries, mean DMFT and SiC scores were higher than the average score of other developing countries. Gaps in dental health service utilization, dental health practices and suboptimal water fluoride levels contribute to poor dental health among school children in Eritrea.

**Electronic supplementary material:**

The online version of this article (doi: 10.1186/s12903-017-0465-3) contains supplementary material, which is available to authorized users.

## Background

Dental caries have been affecting humans since the prehistoric times. Dental caries was ranked as the most common oral condition among 291 diseases between the years of 1990 and 2010 [1]. Worldwide, dental caries is the most prevalent disease of childhood, affecting 60–90% of all children [2].

Majority of dental caries remains untreated due to inappropriate, unaffordable or unavailable dental health services. In most developing countries, the dentist to population ratio is about 1 per 150,000 people, making the provision of dental health services difficult [3]. In Eritrea, the dentist to population ratio is 1 per 303,185 people. This is exacerbated due to generally poor dental health facility infrastructure and low dental health related knowledge and practice in the general public [4].

Globally, dental diseases, mainly dental caries cost 298 billion dollars in direct treatment costs to the global economy, covering up to 4.6% of the global health expenditures. There is also an incurred cost of 144 billion dollars due to loss of productivity; souring the total cost to 442 billion dollars [5].

Moreover, dental problems are a major cause for poor quality of life. Dental caries is the most common oral condition that evokes aesthetic and functional complaints in children. In addition, dental caries is mentioned as the major contributor for the loss of 51 million school hours due to acute dental problems annually [6].

Eritrea is a low-income country located in the horn of Africa. Eritrea has recorded a significant success in achieving the health related Millennium Development Goals in recent years, particularly goals number 4 (reducing child mortality), 5 (improving maternal health) and 6 (combating HIV/AIDS, Malaria and Tuberculosis). Life expectancy in Eritrea has risen from 54.4 in 2000 to 64.7 in 2014 [7]. However, there has been slower progress regarding dental health and dental care activities including prevention and control of dental diseases.

In Eritrea, dental caries is consistently ranked as the first or second most commonly reported disease with the highest morbidity in Out Patient Departments in the country. Dental caries was ranked as the highest morbid disease in the years 2013 and 2014 and second highest reported disease during the years 2011, 2012, 2015 and 2016 [8]. However, as there has been no nationwide research conducted regarding the prevalence of dental diseases, determining the exact burden of dental caries and its associated factors in different settings and groups of people remains a considerable challenge.

The World Health Organization highlights the importance of 12 year olds because it is generally at this age that children finish their primary schooling and hence, in many countries, is the last age at which school based research data can be easily collected. Moreover, all permanent teeth except third molars have erupted by 12 years. Thus, this age was considered to be the age of global monitoring of dental caries for international comparisons and monitoring of disease trends [9].

Therefore, the objectives of this study were to determine the prevalence of dental caries by the DMFT index and significant caries index (SiC) and to assess dental health utilization, practices and their relationship with dental caries among 12 years old students in Eritrea.

## Methods

A cross sectional study was conducted in the central region of Eritrea. It was carried out in two schools (one urban and one rural) among 12 years old students from January to March 2017.

### Sample size estimation

The sample size was determined using single population proportion formula

$$ {n}_1=\frac{z^2p\left(1-p\right)}{e^2} $$

The total sample size was calculated using the following assumptions; proportion (p) of children with dental caries was estimated to be 50% as there was no previous research regarding dental caries in Eritrea, confidence interval 95% (z = 1.96), degree of precision (e) 5%, non response rate of 5%. The final sample was adjusted using the total 12 years old student population in the two schools, yielding a final sample size of 225 students.

### Sampling design

This study employed a purposive sampling design. The selection of one school from an urban and one from a rural community was based on the assumption that data related to each school reflects the situation of the community they serve. First, all subzones in the central region (16 in number) were grouped in to two practical strata (rural and urban).Then convenience sampling was used to select two subzones representing an urban (Asmara) and rural setting (Serejaka subzone, located 12 km north of Asmara).The total list of all middle schools found in the selected subzones were then obtained and simple random sampling technique was employed to select one middle school from each subzone. The total sample size was allocated to the selected schools based on probability proportional to the number of 12 year old students present. Finally, using a list of names of 12 year old students as a sampling frame, a simple random sampling method was employed to select study participants.

### Data collection procedure

Data collection was done by two research team members who were trained to identify dental caries. The training was done by a certified dentist and included both theoretical and practical sessions. The theoretical part included a detailed description of dental caries and numerous illustrations of carious teeth at different locations and of varying degrees of severity. The examiners also took 4 weeks of practical lessons in a dental clinic, where they examined patients and categorized the status of dental caries under the direct supervision of a dentist. At the end of the training session, 20 students were examined by the data collectors independently for dental caries under the supervision of a certified dentist. Inter observer agreement was then calculated using the Cohen’s Kappa statistic (0.82) which indicated “almost perfect agreement”.

Data collection was subsequently carried out using a WHO adopted questionnaire (Additional file 1) for children translated in to the local language (Tigrigna) and a checklist to record the status of each tooth. Written consent was obtained from school directors to conduct the study. Respondents were asked for verbal consent before the interview. Students were examined for dental caries under an artificial light using a dental mirror.

The severity of dental caries was recorded using DMFT and SiC scores. The DMFT index has three parts which are the “D” component, which includes carious teeth, filled teeth with recurrent decay, teeth with only root left, defective filling with caries, temporary filling and teeth with a filled tooth surfaces but with other surface decayed. The “M” component includes teeth that are missing due to caries but it does not include teeth missing for reason other than caries, non erupted teeth or congenitally missing teeth. The last component is the “F” component which includes teeth which have one or more permanent restorations with no secondary (recurrent) caries or other area of tooth with primary caries [9]. The SiC index is the mean DMFT of the top third of the study group with the highest caries score [10].

Instruments used during data collection were sterilized before dental examination and at the end of the data collection period in Biet Mekae community hospital. Disposable gloves and masks were used during the data collection. A sample of water was taken from the water source of both locations and measured for its fluoride content in the National Health laboratory.

Data entry and analysis was done using the Statistical Package for Social Sciences (SPSS ver. 20). Data analysis was performed by applying descriptive statistics and independent sample t-test and chi square tests to assess the relationship between dental caries and related variables. The level of statistical significance was set at *p* < 0.05.

## Results

### Socio demographic characteristics

Out of the 225 participants included in the study, 121 (53.8%) were females. The majority of the respondents (94.2%) were Christian and the Tigrigna ethnic group comprised 96.9% of the study population while Tigre and Saho accounted for 2.7% and 0.4% respectively. More than two thirds of the participants (67.1%) were from rural setting and 32.9% were from the urban setting (Table 1).

**Table 1:** Demographic characteristics of the study participants (*n* = 225)

Demographic variables	*N* (%)
Sex	
Male	104 (46.2)
Female	121 (53.8)
Religion	
Christian	212 (94.2)
Muslim	13 (5.8)
Ethnicity	
Tigrigna	218 (96.9)
Tigre	6 (2.7)
Saho	1 (0.4)
Residence	
Urban	74 (32.9)
Rural	151 (67.1)

### Prevalence of dental caries

More than two thirds 176 (78%) of the respondents had at least one carious tooth during examination or had previous history of carious teeth. Students from an urban setting had higher prevalence of caries than rural (82% versus 76%). However, this was not statistically significant. Similarly, no statistical significance was found between the occurrence of dental caries between female (79%) and male respondents (78%) (Table 2).

**Table 2:** Caries prevalence by gender and residence

	Caries Prevalence		Total	*P* value
	Yes	No		
	N (%)	N (%)	N (%)	
Gender				
Female	95 (79)	26 (21)	121 (100)	0.909
Male	81 (78)	23 (22)	104 (100)	
Residence				
Urban	61 (82)	13 (18)	74 (100)	0.284
Rural	115 (76)	36 (24)	151 (100)	

The mean DMFT value of the participants was 2.50 (±2.21). The decayed (DT) component accounted for 98.3% of the DMFT value with a contribution of 2.44 to the mean DMFT. Missing and filled teeth contributed 0.05 and 0.01 to the mean DMFT respectively (Table 3). Females had DMFT score of 2.56 and males had 2.43, but the difference was not statistically significant. Urban students had a mean DMFT of 2.82 (±2.4) compared to rural students with a mean DMFT of 2.34 (±2.1) which was statistically significant (*p* = 0.05) (Table 4).

**Table 3:** Component of DMFT

Variable	Component of DMFT Score	
	$\left(\overline{x}\pm \mathrm{SD}\right)$	Percentile
Decayed (DT)	2.44 (±2.13)	98.3%
Missing (MT)	0.05 (±0.27)	1.2%
Filled (FT)	0.01 (±0.2)	0.4%
Total	2.50 (±2.21)	100%

**Table 4:** DMFT, DMFT of the first molar and significant caries index by age and residence

Score	Gender		Residence		
	Male	Female	Urban	Rural	Mean score ± SD
DMFT $\left(\overline{x}\pm \mathrm{SD}\right)$	2.43 (2.14)	2.56 (2.27)	2.82 (2.4)	2.34(2.1)	2.50(2.21)
*P* value	0.738		0.05		
First molar DMFT	1.50 (1.41)	1.58 (1.3)	1.72 (1.35)	1.46 (1.37)	1.55(1.36)
*P* value	0.386		0.697		
Significant caries index	5.07 (1.76)	4.91 (2)	5.73 (2.097)	4.66 (1.74)	4.97 (1.9)
*P* value	0.948		0.012		

The first molar was the most affected tooth as it alone had a DMFT value of 1.55 (±1.36). Female students had higher DMFT of the first molar of 1.58 whilst males had a value of 1.5. DMFT value of 1.72 (±1.35) was scored from students residing in the urban school, which is higher than rural students with a score of 1.46 (±1.37). Both gender and residence analyses were not statistically significant as they had *p* value of 0.386 and 0.697 respectively (Table 4).

A mean score of 4.97(±1.9) was recorded for the significant caries index. Males had higher SiC score (5.07 ± 1.76) than females (4.91 ± 2.00). A statistically significant difference was observed in SiC scores between urban (5.73 ± 2.09) and rural students (4.66 ± 1.74).

### Previous experience of dental pain and utilization of dental health facility

Almost half (48%) of the respondents reported that they had previously experienced dental pain with varying frequency. Moreover, more than half (69%) of the study participants had never visited a dental facility, while 3% who had done so had not visited in the 12 months prior to the study. Out of those who had visited a dental health facility, visits for regular checkup was cited by only 6.6% of the students. Most visits (82%) were due to pain of teeth, gum or mouth. Of those who had not visited a dental health facility, 82% reported they had no reason for not utilizing one while “fear of painful procedures” was mentioned by 5% of the respondents.

### Dental hygiene practice and tools utilized

More than one quarter (28.1%) of the study participants said they cleaned their teeth “once a day” and 19.1% twice daily. The most common tools used were brushes and local “chew sticks” with reported utilization by 76.4% and 78.7% respectively. Three quarters (75%) of the study participants reported utilization of tooth paste when brushing. Moreover, the majority of the students (85%) claimed that they cleaned their teeth unassisted while 9% reported they received assistance from their mother.

### Perceived status of children’s teeth and gum

More than half of the study participants (52%) said they thought their teeth were ‘fair’ and 32% said they were ‘good’. 11% of the study participants considered their teeth to be ‘bad’. Half of the study participants (51%) said their gums were good while only 4% admitted that their gums were bad.

### Fluoride sources

Water in the urban area had a fluoride content of 0.28 and in the rural area 0.53 Parts per million (ppm). Since the optimum fluoride content in water for the reduction of dental caries is 1 ppm, the level of water fluoridation was sub-optimal.

## Discussion

With a prevalence of 78%, dental caries was a common public health problem among 12 year old students in Eritrea. This is similar to the 77% prevalence reported in India [11] but was higher than rates described from Nigeria (13.9%) [12], Kashmir (25%) [13], Sudan (30.5%) [14] or Tamil Nadu India (40%). [15]. These different prevalence figures could be attributed to many factors including socio cultural differences, study setting, sample size, dietary behaviors and differences in knowledge, attitude and practice regarding dental hygiene.

In the present study, only 19.1% of the study respondents said they cleaned their teeth twice daily. Moreover, the majority of the participants reported they clean their teeth unassisted which may mean that they were not cleaning their teeth effectively. The majority of the students (96.4%) cleaned their teeth using different tools. Tooth-brush (76.4%) and local chew-stick (78.7%) were the most commonly utilized. These figures are higher with other studies [16, 17] that showed that tooth brush and tooth paste being the most common means of maintaining oral hygiene. In Eritrea, many species of trees are used as chew stick. The selection of these species usually depends on personal preference and availability in the neighborhood. However, the most common selection method is based on perceived power of cleaning of the tree species. Some of the usually used species in Eritrea include *Olea europea*, *Euclea schimperi*, *Rumex nervoses* and *Cadaba farinosa* [18]. A chew stick from these trees is cut and prepared in a suitable design or shape and is used for cleaning the teeth without any tooth-paste.

As in Kenya and Nigeria, where no dental visit was reported by 46.7% [19] and 80% [12] of the study participants respectively, more than half (69%) of the participants in the present study said that they have never visited a dentist in their life. Among the students who had visited a dentist, more than two thirds (82%) went due to dental pain, which was similar to studies done in Iraq (71.2%) [20] and Kenya (73.4%) [19]. Thus, poor utilization of dental health facilities for prevention and promotion of dental health was a potential contributor to the high prevalence of dental caries.

The total mean DMFT of the study participants was 2.50 (±2.21), which is very high when compared to the studies done in Kenya (0.42) [19], Nigeria (0.14) [12] and Sudan (0.42) [14]. The decayed component accounted for 98.3% of the DMFT value as it had a 2.44 contribution to the mean DMFT. This clearly indicates under-utilization of dental health facilities, unhealthy dietary habits and gaps in the knowledge, attitude and practice of dental hygiene.

Since the caries distribution is generally observed to be skewed, the significant caries index addresses individuals with the highest caries scores. The mean SiC score in this study was 4.97. This figure is high compared to the goal set by WHO which suggested that countries should limit the SiC score to be below 3 by the year 2015 [10].

In this study, water fluoride content in both localities had fluoride concentration of 0.28 ppm in urban and 0.53 ppm in rural setting. Water source in both locations was thus below optimum level for protective effect against dental caries. This low water fluoride level could be a further contributor to the high prevalence of dental caries. A similar result was found in a study done in Iraq, where the water fluoride level was 0.14 ppm [20].

This study has assessed the prevalence and factors associated with dental caries in 12 year old children in Eritrea. Due to feasibility and administrative reasons, this study employed a purposive sampling design. Hence the results, even though they may shed light to the current situation, may not be generalized to the whole 12 years old student population in Eritrea.

## Conclusion

Dental caries is a common public health problem among 12 years old Eritrean students with the mean DMFT and significant caries index (SiC) being 2.50 and 4.97 respectively. These values are considerably higher compared to reports from other, mainly African, developing countries. The suboptimal water fluoride level along with poor dental service utilization is a major challenge to children’s dental health. There was also a gap concerning dental health practices. Only half of the respondents practiced dental hygiene on a daily basis using different means, with tooth-brush and chew stick being the most commonly tools utilized.

## Additional file

Additional file 1: Oral Health Assessment form for Children. The instrument is designed by the WHO for oral health assessment for children. (DOCX 107 kb)

## Abbreviations

DMFT: Decayed, missing, filled teeth
DT: Decayed tooth
HIV/AIDS: Human immune deficiency virus/Acquired immune deficiency syndrome
PPM: Parts per million
SiC: Significant caries index
SPSS: Statistical Package for Social Science
WHO: World Health Organization
