# Supplementary figures and images for: Prevalence of dental caries and associated factors among 12 years old students in Eritrea
Source: BMC Oral Health. 2017 Dec 29;17:169. doi: 10.1186/s12903-017-0465-3 (PMC5747091; doi:10.1186/s12903-017-0465-3)

World Health Organization

Oral Health Assessment Form for Children


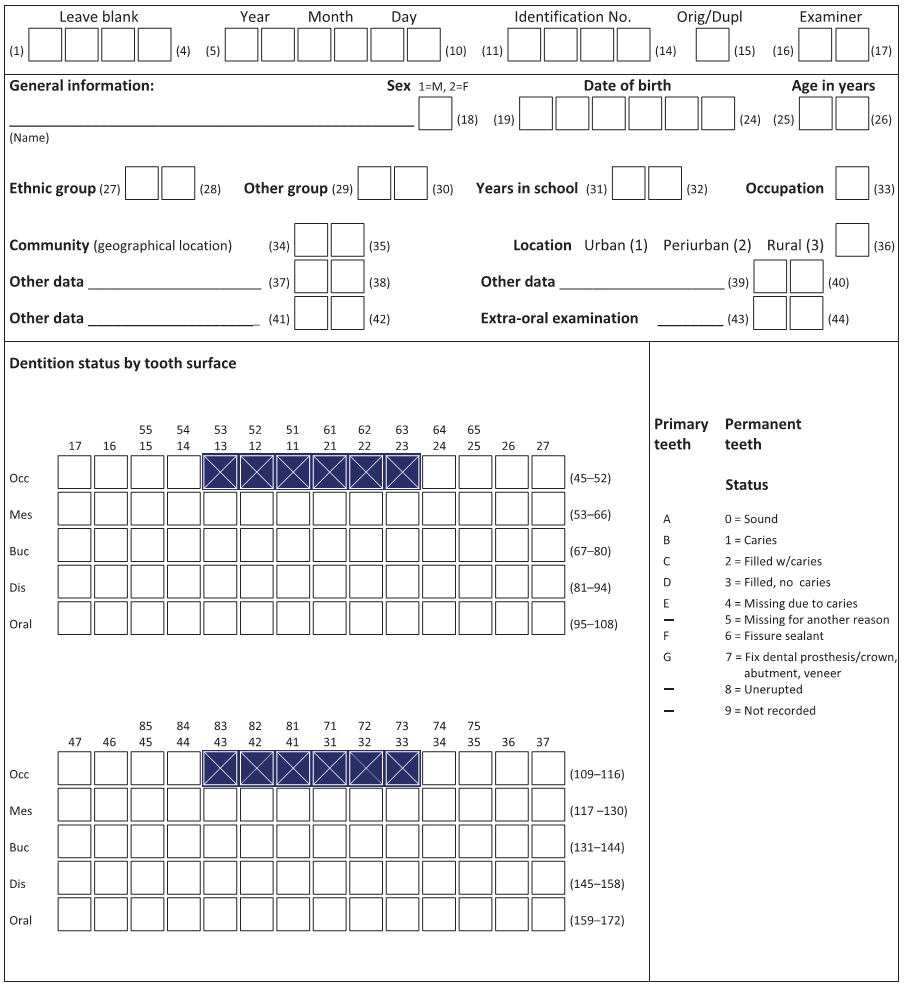

Supplement: Additional file 1: — Oral Health Assessment form for Children. The instrument is designed by the WHO for oral health assessment for children. (DOCX 107 kb) [file 12903_2017_465_MOESM1_ESM.docx]
